# Industrial noise: A new stimulus for dental pulp aging? Qualitative and quantitative analysis in Wistar rat teeth–A pilot study

**DOI:** 10.3389/froh.2022.859664

**Published:** 2022-09-15

**Authors:** Maria Alzira Cavacas, Carolina Doroteia, Ana Margarida Leonardo, Luís Proença, Vítor Tavares

**Affiliations:** ^1^Centro de Investigação Interdisciplinar Egas Moniz (CiiEM), Almada, Portugal; ^2^Anatomy Department, Instituto Universitário Egas Moniz, Almada, Portugal; ^3^Histology and Embryology Department, Instituto Universitário Egas Moniz, Almada, Portugal; ^4^Instituto Universitário Egas Moniz, Almada, Portugal; ^5^Quantitative Methods for Health Research, Centro de Investigação Interdisciplinar Egas Moniz (CiiEM), Egas Moniz, Almada, Portugal

**Keywords:** histology, industrial noise, pulp cell density, pulp-dentin complex, Wistar rat, qualitative and quantitative analysis

## Abstract

Morphological changes induced by industrial noise have been experimentally observed in several organs. This study aims to characterize the effect of industrial noise on the pulp cell density of Wistar rats from a histological point of view, through qualitative and quantitative analysis. The histologic sections were observed over the optical microscope and photographed using 10x and 40x magnifications and analyzed using an image processing software. They refer to a group of animals exposed to industrial noise for 3 months and for 7 months (EG) and another group of animals kept in silence for control (CG) during the same periods. There was a significant decrease in pulp cell density in teeth of the animals exposed for 3 months when compared to control teeth group (*p* = 0.01). However, in the group exposed for 7 months, no statistically significant differences were found (*p* = 0.66). Even so, we found an evident relationship between exposure to industrial noise and teeth morphological changes. The observed changes are similar to the modifications found on aged teeth. Therefore, this study places industrial noise as an aggressive stimulus that can cause a reaction of the pulp-dentin complex with morphological changes compatible with premature aging of the tooth.

## Introduction

Noise exposure is an important health issue because of the continuous industrial growth, considered today as the second risk factor for public health–being airborne particulate matter the most significant environmental risk factor [1–3]. The World Health Organization published its most recent noise pollution guidelines for Europe in 2018 [4]. This publication states that “noise is an important public health issue; it has negative impacts on human health and well-being and is a growing concern.” The World Health Organization Regional Office for Europe has developed guidelines which provide recommendations for protecting human health from exposure to environmental noise and are essential to drive policy action that could protect communities from the adverse effects of noise.

Industrial noise is characterized by high intensity and a wide spectrum of wavelengths that includes low-frequency noise. This one is an agent of disease that often is not taken seriously in noise protection procedures due to the fact that the human ear is less attuned to the acoustic phenomena within the lower frequencies (<500 Hz), and it is not able to perceive (<20 Hz), only in the form of vibrations [5, 6].

Although the human ear cannot detect lower frequencies, noise is considered a stress factor that involves several negative effects on multiple organs and systems when exposed to it [2, 7]. Most investigations focus on auditory effects; however, the effects of sound exposure can go beyond hearing loss [8, 9]. The non-auditory effects of noise exposure can change the normal procedures of other organs and systems [10], including: degenerative cellular changes, stomach vascular injury [8], increased connective tissue in the liver [11], swelling of the renal glomerulus [12] fibrosis in the cardiac muscle [13], increased perivascular tissue of coronary arteries with significant periarterial fibrosis [14], induction of coronary perivascular fibrosis that differs under corticosteroid administration [15], increased in atrial interstitial fibrosis and a decreased in connexin 43 in rat hearts [16], increased risk of hypertension [17], increased in myocardial infarction and strokes [18] and changes in the adrenal cortex [19]. In the oral cavity, the authors described a morphological and functional change in the parotid gland [6], periodontal injury [20], and dental wear [21, 22].

Studies by Cavacas et al. [21] showed that the teeth of rats exposed to industrial noise had a band-like structure between the pulp and dentin in the upper chamber, which is probably tertiary dentin, produced in response to noise exposure by odontoblast-like cells. These changes may be related to the direct impact of sound pressure or to an adaptive response of the pulp-dentin complex related to stress, since excessive stress results in hyperactivity of the masticatory muscles, as seen in parafunctional activities, particularly bruxism [23–27].

The pulp-dentin complex is a notable symbiose: dentine and pulp are two tissues strongly related [28]. Both suffer deteriorating changes due to aging, caries, injuries, cavity preparation, adhesive techniques, and tooth wear. Nanci [28] and Daud et al. [29] describe that pulp tissue suffers a reduction in the pulp chamber by continuous formation of dentin, an obliteration of the pulp canal, which can result in reduced vascularity, structural changes in blood capillaries, presence of fibrous bundles resulting from a change in the distribution of collagen fibers and a reduction of pulp cell density. The dentine presents a complete tubule closure and an increase of sclerotic dentin formation, decreasing dentin permeability and making it more brittle. Daud et al. [29] and Murray et al. [30] state that dental aging leads to a reduction in dental pulp density and changes in cell morphology which compromise the repair capacity of the pulp during an injury.

Several researchers have described similarities between rat teeth and human teeth at the histological and physiological level in shape and function. In addition, the teeth of these animals appear to be an adequate model for the study of age-related changes in the cells of the pulp-dentin complex, since it would be difficult to obtain an adequate number of samples of human teeth from different age exposed to industrial noise [30].

There are several studies on the health impact of occupational and environmental noise exposure. However, there are still few studies that focus only on health impacts from low-frequency noise, especially industrial noise [1]. From the changes reported in the studies above, it is clear that the stomatognathic system is also affected by the effects of prolonged exposure to noise. So, with this study we intend to characterize the effect of industrial noise on circumpulpar dentin and dental pulp of Wistar rats from a morphological, specifically histological point of view, through a qualitative and quantitative analysis, by evaluating the pulp cell density.

## Materials and methods

This work was undertake following the materials used in previous studies [21, 22] and it was conducted as a pilot study.

### Animals

For the present study, a convenience sample of 50 Wistar rats (Charles River Laboratories España SA, Spain) were included: 25 males and 25 females. The Wistar rats were placed in cages, where they could move freely. More than two animals of the same sex were never placed in each cage. Rats were fed standard rat food and they had free access to water. Daily cycles were maintained with 12 h of light and 12 h of darkness. The animals were treated in accordance with the EU Commission on Animal Protection for Experimental and Scientific Purposes (86/609/EEC) and with the Portuguese legislation for the same purpose (Decree-Law No. 197/96).

### Noise exposure

The noise of a textile factory in northern Portugal was used to simulate the occupational environment to which workers are subjected. The noise present in the workplace was recorded and reproduced using an electroacoustic set based on a computer system, with a DT2823 data acquisition card and SB Live 5.1 card, a B&K 4165 microphone with preamplifier, a 2-channel amplifier, a “subwoofer” and 16 monitor-type speakers. The software was designed using the LabVIEW system. The processing of sound signals was done offline, applying the LabVIEW and Matlab systems. The characterization of the frequencies and amplitude of the signals was carried out in all samples.

The rats were divided into two groups with the same sex number: 10 animals were kept in silently for control (Control Group–CG) and half of them were sacrificed at 3 months and the other half were sacrificed at 7 months.

The remaining 40 animals were exposed to industrial noise from 1 to 7 months, at different periods of time, simulating a working time schedule (8 h/day, 5 days/week, and weekends in silence). These 40 rats (Exposed Group–EG) were divided into two subgroups of 20 animals each and exposed to industrial noise for 3 months (480 h) and 7 months (1,120 h).

### Histological model

After the exposure to industrial noise, the animals were sacrificed with an intraperitoneal injection of Ketamine. The dental arches of all animals were carefully observed.

Molars from each group were selected for surgery removal. The extraction of molar teeth was performed according to the surgical principles applied in Dentistry. A syndesmotome was used in the 295-3 (ASA DENTAL) and very gentle forces were applied to dislocate the teeth, considering their small size and the fragility of the roots.

The teeth were fixed in 10% buffered formalin for observation under light microscopy. Fragments fixed in 10% formalin were decalcified in 10% nitric acid for 48 h. After complete processing, the teeth were included, cut longitudinally in the mesio-distal direction, and stained with Hematoxylin and Eosin staining.

### Experimental protocol

**Figure 1:**
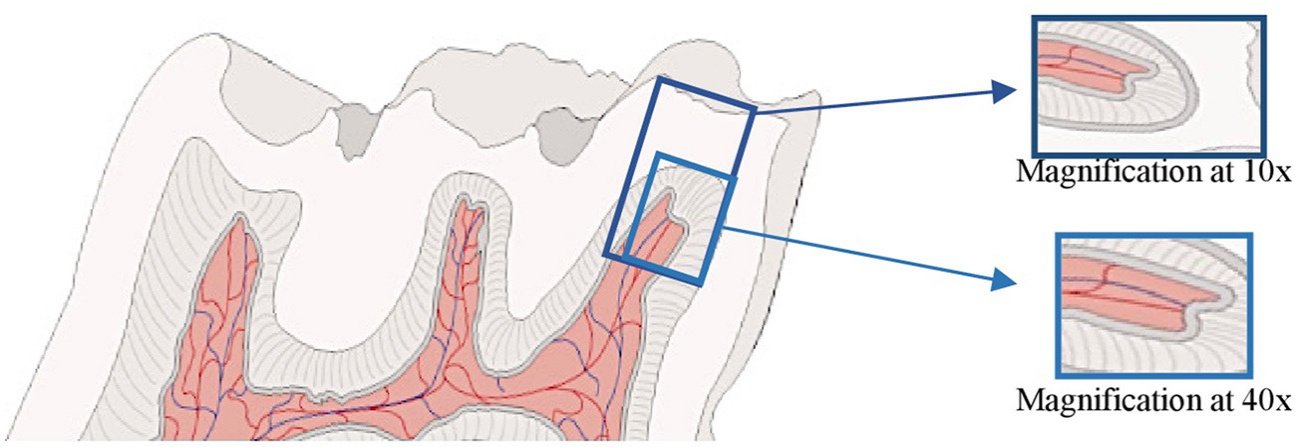
Illustration of a Wistar rat's molar analyzed: pulp horn and respective cusp at 10x magnification and pulp horn chamber at 40x magnification.

The samples were observed with optical microscopy and photographed always by the same observer. Control and industrial noise exposure blades with higher quality were selected. For each period, we considered the same molar cusp for both groups (control and exposure) and analyzed the respective region of the coronary pulp with a magnification of 10x and 40x (Figure 1).

For all samples, five regions were considered–dentin, pre-dentin, odontoblastic layer, subodontoblastic layer, and fibroblasts—to compare the morphology, specifically the histology of these regions: comparing the exposure samples from each group with the respective control.

**Figure 2:**
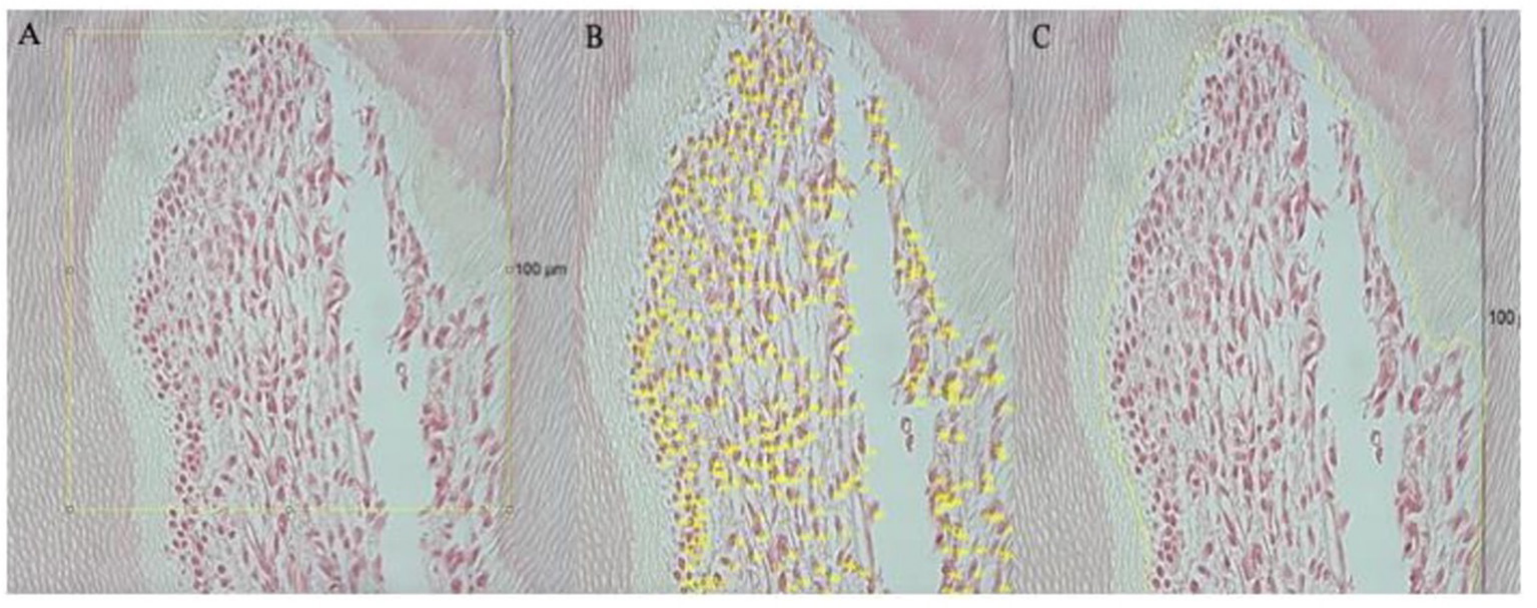
Representative of the tools used in Image J. Yellow line represents the area that was measured and quantified; **(A)** shows the pulp region analyzed, based on the 100 μm scale; in **(B)** it is possible to observe the manual counting of the cells; **(C)** shows how the area was measured, covering only the cells that were quantified.

Regarding the quantitative analysis of pulp cells, the photographed samples were analyzed and quantified using the ImageJ software (1.53 a), starting with the measurement of 100 μm, in a straight line, from the first cell (odontoblast) of the apex's cusp to the base, thus establishing the region to be quantify (Figure 2A). Then, manual cell counting was performed (Figure 2B), and the area corresponding to the quantified cells was calculated (Figure 2C). To minimize errors, eight images *per day* were analyzed, by two independent observers and in duplicate. All disagreements were resolved through discussion with a third observer.

### Included and excluded criteria

The samples included were those where the pulp region was visible, with a longitudinal section.

The samples excluded from the study were those with an oblique cut.

### Statistical analysis

Statistical analysis was performed with the IBM SPSS^®^ Statistics (v. 26) software. Data was analyzed by means of descriptive and inferential methodologies. Data adequation to normality was evaluated by the Shapiro-Wilk test. Due to the characteristics of the outcome variable (non-normal distribution and small sample size), the Mann-Whitney test was used to compare it between groups. The inferential analysis was performed at a 5% significance level.

## Results

Histological sections showed differences between the cusps of the molars exposed to industrial noise and the cusps of the molars of the control groups. The results were divided into qualitative observations and quantitative analyses. Within each of these analyses, the results at the 3^rd^ and 7^th^ months of exposure were demonstrated, as well as the corresponding controls.

### Qualitative analysis

#### Third month

In the qualitative analysis, comparing the control samples to the exposure samples at 10x magnification, there is a rounded coronary pulp with a greater distance to the respective cusp due to a thicker dentin layer. However, the exposure sample at 10x submitted to a longer time of industrial noise shows a decrease in distance from the coronary pulp to the cusp, maybe due to an increase of dental wear.

**Figure 3:**
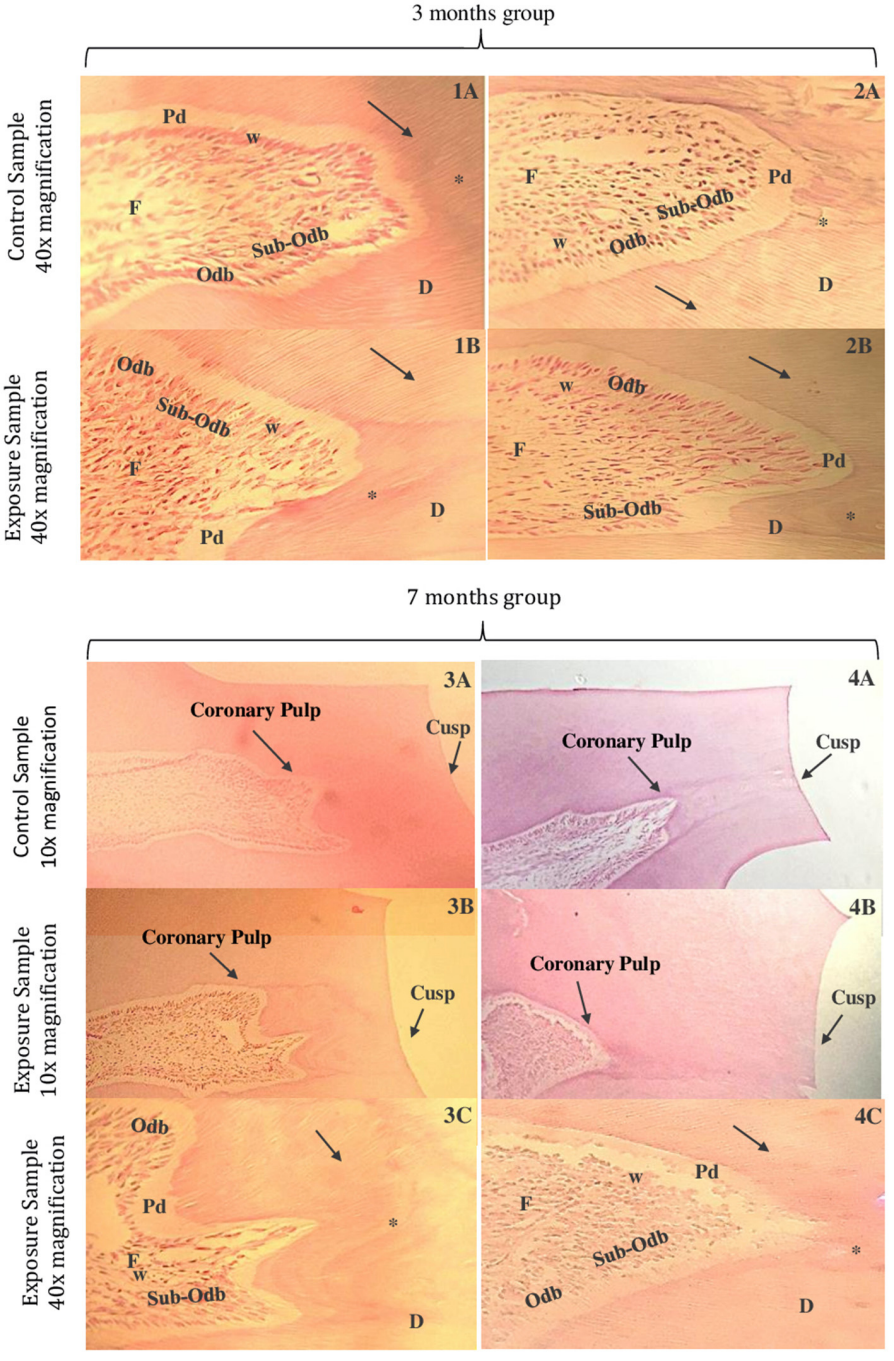
Microphotographs of cross-sections of Wistar rat teeth. Third month control group samples at 40x magnification **(1A,2A)**. Third month exposed group samples at 40x magnification **(1B,2B)**. Seventh month control group samples at 10x magnification **(3A,4A)**. Seventh month exposed group samples at 10x magnification **(3B,4B)**. Seventh month exposed group samples at 40x magnification **(3C,4C)**. Dentin (D), pre-dentin (Pd), dentin tubules (arrow), odontoblastic layer (Odb), subodontoblastic layer (Sub-Odb), *Weil* zone (w), fibroblasts (F), and tertiary dentin incremental lines (*) (HE).

In a comparation between the control samples (Figures 3–1A) and the exposure samples (Figures 3–1B), both at 40x magnification, we can observe in this last one: the presence of tertiary dentin incremental lines and the dentin tubules are less visible; pre-dentin layer is less thick, and the *Weil* zone seems normal; odontoblastic layer suffers a decrease in thickness; subodontoblastic layer is present and seems in its normal morphology; fibroblasts seem more separated within themselves.

Comparing the control sample in a 40x magnification of Figures 3–2A and the exposure sample also in 40x of Figures 3–2B, it was verified: the dentin layer containing tertiary dentin incremental lines and dentin tubules is less visible; pre-dentin layer appears to be less thick and the odontoblastic layer seems to be less visible, with the odontoblasts more separated; subodontoblastic layer seems disorganized and its cells in less quantity; *Weil* zone seems irregular and fibroblasts appear to decrease in number, with their extensions shortened.

#### Seventh month

The major difference of coronary pulp observed between the one from the control sample at 10x magnification (Figures 3–3A) and from the exposure samples (Figures 3–3B) was that the coronary pulp from the last one appears to be more far from the respective cusp. In the exposure sample at 40x magnification (Figures 3–3C), compared to the control sample at the same magnification, it seems that: the thickness of the dentin has significantly increased at this period; tertiary dentin incremental lines in a higher number and the dentin tubules less visible; pre-dentin layer appears to be significantly less thick and *Weil* zone seems to be less visible; odontoblastic layer seems to have less quantity of cells and subodontoblastic layer appears to be less visible; fibroblasts seems to decrease in number.

**Figure 4:**
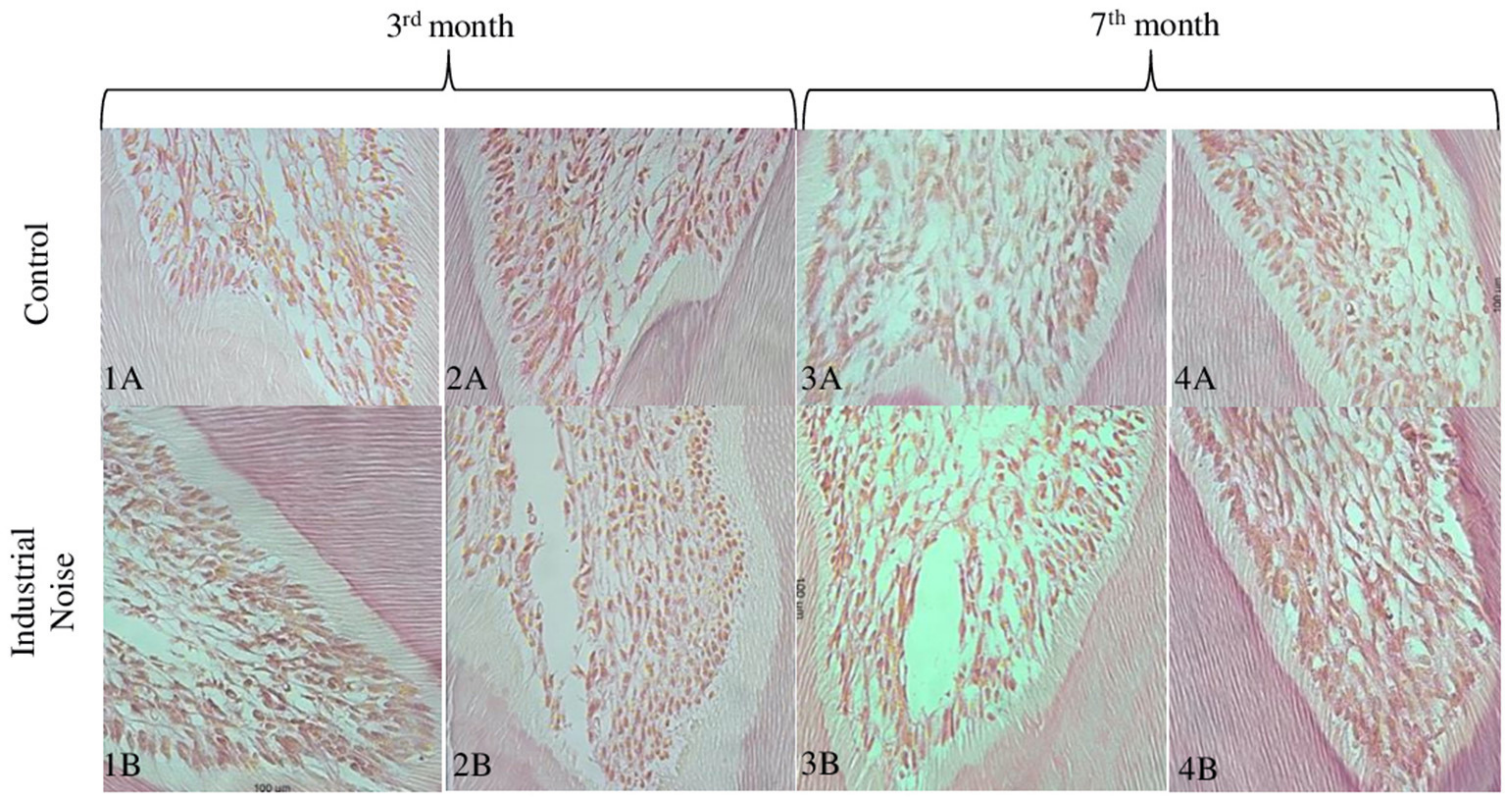
Microphotographs of cross-sections of Wistar rat teeth, where it is possible to observe the cells in the coronary portion of the dental pulp. Representative samples from the 3rd and 7th months of control **(A)**, and from the 3rd and 7th months of exposure **(B)** (HE, 40X).

There is a significantly change in the coronary pulp form of the samples submitted to a longer time of industrial noise–when comparing the control sample (Figures 3–4A) to the exposure sample (Figures 3–4B), both at 10x magnification: in the last one, the coronary pulp form appears rounded and more far away from the respective cusp which make us assume that is associate to the increase thickness of dentin–it can be seen an area with more contrast, which corresponds to tertiary dentin.

In the exposure sample at a 40x magnification of (Figures 3–4C), it appears that it is more difficult to see the dentin tubules; pre-dentin layer is almost invisible, and the *Weil* zone is nearly absent; an odontoblastic layer disorganized and a sub-odontoblastic layer not easy to find, maybe because cells suffered a differentiation to odontoblastic cells; fibroblasts appear to be in a large number and less disorganized.

### Quantitative analysis

#### Third month

In Figure 4 can be seen the control group rat dental pulp (Figures 4–1A,2A) and the rat's exposed to noise pulp (Figures 4–1B,2B). There seems to be a marked difference in pulp cell density, with a decrease in the number of cells per unit area (μm^2^) in the group exposed to industrial noise, when compared to the control group (Table 1). Quantitative analysis confirms that there are statistically significant differences in pulp cell density between the exposed group and the control group (*p* = 0.01) (Table 1), with a smaller number of cells per unit area (μm^2^) in the exposed group.

**Table 1:** Pulp cell density measures for the control group (CG) and the exposed group (EG) at the 3^rd^ month and the 7^th^ month.

	**3**^**rd**^ **month**			**7**^**th**^ **month**		
	**Mean (SD)**	**Min.-Max**.	*p* *	**Mean (SD)**	**Min.-Max**.	*p* *
CG	7.54 (0.35)	7.08–7.88	0.01	7.37 (1.62)	6.07–10.30	0.66
EG	5.78 (0.50)	5.11–6.24		6.63 (1.24)	5.03–8.27	

Values presented as mean (standard deviation, SD).

^*^ Mann-Whitney test.

#### Seventh month

Comparing Figures 4–3A,4A (control) with Figures 4–3B,4B (exposed), it is observed that although there may be a decrease in pulp cell density in the group exposed to noise, this decrease seems to be less pronounced when compared to the previous months mentioned (Table 1). There seems to be a gradual increase in similarity between the control group and the exposed group.

Through statistical analysis, it was found that there are no significant differences between groups (*p* = 0.66).

## Discussion

Several studies have pointed out noise as an aggressive agent that causes several changes in biological tissues, namely at the level of the stomatognathic system [6, 8, 10–18, 20–22]. Exposure to noise pollution has increased, in contrast to other pollution factors [31]. Thus, it becomes important to determine and understand the possible effects of these on health, especially in the pulpo-dentin complex.

Our qualitative analysis corroborates the results of previous studies of our group that showed an apparent increase in tertiary dentin and a decrease in the pre-dentin layers [21, 22]. Moreover, tertiary dentin is found near the pulp chamber because it is formed by odontoblastic cells as a response to the stimulus (industrial noise) to protect the pulp [32, 33].

The odontoblastic layer appears to suffer a decrease in its density, maybe because odontoblasts suffer a reduction in size and reorganization, leading to a less capacity for an aggression response [32–35].

Near to this layer, we can find the *Weill* zone that seems to decrease in thickness, when related to the pulp cells rearrangement due to the stimulus [22].

Subodontoblastic cells also appear to suffer a density decrease due to the differentiation into odontoblast-like cells, which have restored activity [29, 32–34].

Fibroblasts seem to decrease their number on the 3rd month [29, 32]. However, on the 7th month it appears that they suffer an increase, maybe related to their function: these pulp cells are responsible for pulp matrix maintenance with the production and turnover of collagen, which is important to the dental tissue repair [32, 33].

In this investigation, there was a decrease in cell density in teeth exposed to noise compared to control teeth. However, it was observed that this decrease is not constant over the months. According to the results obtained, there appears to be a marked decrease in cell density in the first 3 months. Between the 4^th^ and the 7^th^ month, there is a gradual increase in similarity between the control group and the exposed group.

This decrease in the number of cells may be related to a response of the organism to industrial noise-which may occur due to psychological stress and/or to the physical vibration of tissue structures-or to an adaptive response of the organism and the pulp-dentin complex to adapt to the narrowing of the pulp chamber, which occurs through the deposition of tertiary dentin [33]. This narrowing has been associated with environmental influences (such as industrial noise) and aging.

In the literature, several age-related changes in the pulp-dentin complex are described. The dental pulp decreases in volume, becomes more fibrous, less vascularized, and less innervated, and cell density decreases [33, 34], corroborating studies that show a reduction in the total number of dental pulp cells with increasing age [29, 32–34].

According to the results obtained through statistical analysis, there are significant differences in pulp cell density between the exposure and control groups on the 3rd month (*p* = 0.01). The decrease in cell density verified is in line with several studies that showed that the tooth responds to aggressive agents through the pulp-dentin complex [30, 32–35]. However, on the 7th month of exposure, there were no statistically significant differences between the exposed group and the control group (*p* = 0.66). These results can be explained by the adaptive response of the pulp tissue of the tooth to the aggressor or to stressful conditions, as well as by the pulp physiological aging of the control tooth.

As a limitation of the study, we can point out the fact that it was aimed as an exploratory study, so it was not supported by an *a priori* sample size calculation. Also, the fact that we limited our observations to the histological modifications induced by industrial noise in the pulp-dentin complex and dental pulp (its density and cell morphology) and can only extrapolate these results to humans with limitations. In an identical but prospective study, it will be interesting to differentiate the quantification of dental pulp cell populations.

## Conclusion

The main purpose of this study was to evaluate the changes in pulp cell density in teeth exposed to an external aggressor. As reflected in the general objective of the present investigation, there were qualitative and quantitative changes in the pulp-dentin complex of Wistar rat teeth exposed to industrial noise, especially a decrease in pulp cell density.

In summary, we found a relationship between exposure to industrial noise and teeth morphological changes. This study places industrial noise as an aggressive stimulus capable of causing a reaction of the pulp-dentin complex, with morphological changes compatible with premature aging of the tooth.

Although there are several histological and physiological similarities between rat and human molars, these results can only be extrapolated to humans with limitations.

## Data availability statement

The original contributions presented in the study are included in the article/supplementary material, further inquiries can be directed to the corresponding author.

## Ethics statement

The animal study was reviewed and approved by the animals were treated in accordance with the EU Commission on Animal Protection for Experimental and Scientific Purposes (86/609/EEC) and with the Portuguese legislation for the same purpose (Decree-Law No. 197/96).

## Author contributions

MC: conceived and designed the analysis, collected the data, performed the analysis, and wrote the paper. AL and CD: collected the data and analysis tools, performed the analysis, and wrote the paper. VT: review paper. All authors contributed to the article and approved the submitted version.

## Conflict of interest

The authors declare that the research was conducted in the absence of any commercial or financial relationships that could be construed as a potential conflict of interest.

## Publisher's note

All claims expressed in this article are solely those of the authors and do not necessarily represent those of their affiliated organizations, or those of the publisher, the editors and the reviewers. Any product that may be evaluated in this article, or claim that may be made by its manufacturer, is not guaranteed or endorsed by the publisher.
